# Targeting the gut microbiota to promote motor rehabilitation after spinal cord injury: mechanisms, evidence, and translational challenges

**DOI:** 10.3389/fneur.2026.1946470

**Published:** 2026-10-01

**Authors:** Zhi Chen, Zhenzhen Gao, Zhiwen Zhu

**Affiliations:** 1 Department of Anorectum, The First Affiliated Hospital of Zhejiang Chinese Medical University (Zhejiang Provincial Hospital of Chinese Medicine), Hangzhou, China; 2 Department of Rehabilitation Medicine, The First Affiliated Hospital of Zhejiang Chinese Medical University (Zhejiang Provincial Hospital of Chinese Medicine), Hangzhou, China

**Keywords:** evidence appraisal, gut microbiota, gut-spinal cord axis, motor recovery, narrative review, short-chain fatty acids, spinal cord injury, translational medicine

## Abstract

Spinal cord injury (SCI) is a devastating central nervous system trauma that frequently results in permanent motor dysfunction. Over the past decade the gut microbiota has emerged as a potentially modifiable contributor to secondary injury and neural repair, giving rise to the concept of the gut-spinal cord axis. In this narrative review we synthesize current understanding of how SCI-associated gut dysbiosis may relate to the recovery of motor function, focusing on immune modulation, microbial metabolites such as short-chain fatty acids and bile acids, and gut-central nervous system signaling. Throughout, we deliberately separate evidence obtained in SCI models or in people with SCI from mechanistic evidence generated in other diseases or experimental systems, because conflating these sources is a recurring source of over-interpretation in this field. We then summarize microbiota-targeted interventions studied in animal models-probiotics and prebiotics, fecal microbiota transplantation (FMT), antibiotics and bacteriophages, and direct supplementation with microbial metabolites-and appraise the small, predominantly observational human literature. Several of these interventions improve locomotor outcomes in rodent SCI models, but, with rare exceptions, the causal chain from a microbial change to a functional improvement has not been demonstrated within a single SCI study, and no randomized controlled trial in people with SCI has used motor function as its primary endpoint. We conclude by dissecting the principal translational barriers-inter-individual variability in microbiota composition, uncertain intervention timing and duration, unresolved safety questions, and the absence of standardized pre-clinical-to-clinical pipelines-and by outlining the study designs and reporting standards required to move this field from association toward causation.

## 1 Introduction

Spinal cord injury (SCI) is a devastating neurological condition characterized by disruption of neural pathways within the spinal cord, producing profound motor, sensory and autonomic dysfunction. Its epidemiological burden is substantial: thousands of new cases occur annually, and chronic disability-including paraplegia and tetraplegia-is common, with major consequences for quality of life and considerable socioeconomic cost (1, 2). Motor deficits arise from the primary mechanical insult and are compounded by secondary injury cascades, including ischemia-reperfusion injury, inflammation and neurodegeneration, which drive demyelination, axonal degeneration and neuronal apoptosis and thereby constrain axonal regeneration and functional recovery (3, 4). It is useful to separate two therapeutic domains whose limitations have different causes. Regeneration-directed strategies-cell transplantation, biomaterial scaffolds, and neurotrophic-factor or gene delivery-are limited by the inhibitory lesion environment, the low intrinsic regenerative capacity of adult axons, and unresolved questions of dose, timing and delivery (5). Conventional functional rehabilitation, comprising task-specific physical and occupational therapy, is limited instead by training dose, access, and the physiological ceiling imposed by the injury itself; the cardiovascular stress actually achieved during routine inpatient rehabilitation is modest in many patients and may be insufficient to drive the intensity-dependent neuroplastic effects that experimental work predicts (5, 6). We therefore use the term “plateau” descriptively rather than as a formal claim: despite incremental advances in acute management and rehabilitation, a large proportion of people with SCI do not regain independent walking. Distinguishing these two sets of constraints matters because they imply different therapeutic targets. Regeneration-oriented approaches aim to rebuild the anatomical and physiological substrate for conduction, whereas rehabilitation aims to exploit whatever substrate survives; interventions that reduce secondary injury, systemic inflammation, or both are plausibly relevant to each. Moreover, when asked directly, people living with SCI rank restoration of motor function, together with bladder, bowel and sexual function, among their highest priorities, above several secondary complications that have historically dominated the research agenda (7). This mismatch motivates the present review: we ask whether, and on what evidence, the gut microbiota can be engaged to support motor recovery specifically.

Over the past decade the gut microbiota has emerged as a critical modulator of host physiology, influencing immune regulation, metabolism and neural function. The gut microbiome-the largest “microbial organ” of the body-produces a wide array of metabolites and signaling molecules that can influence the central nervous system through what is now termed the gut-spinal cord axis, a bidirectional communication system operating through immune, neural, endocrine and metabolic routes (8, 9). After SCI, both clinical and rodent studies consistently report compositional shifts in the gut microbiota, a state referred to as dysbiosis. Reported changes include lower relative abundance of taxa associated with short-chain fatty acid (SCFA) production and barrier support-for example Bacteroides and butyrate-producing members of the Lachnospiraceae and Ruminococcaceae-together with increases in taxa that expand under inflammatory or dysbiotic conditions, such as Enterobacteriaceae and Veillonellaceae (10–12). Two caveats are essential and are developed throughout this review. First, taxonomy is frequently reported at inconsistent ranks, and the direction of change is not uniform across studies. Prevotellaceae, for example, is a family that contains both commensal and opportunistically pathogenic members, and Prevotella is a single, taxonomically and functionally heterogeneous genus within it; the same is true of *Lactobacillus*, a genus of more than 200 species with strain-specific and host-context-dependent effects. Statements that treat *Prevotellaceae, Prevotella* or *Lactobacillus* as uniformly beneficial or uniformly pathogenic are therefore not supportable, and throughout this review we specify the rank and, wherever the primary source allows, the species or strain (11, 12). Second, most human findings are cross-sectional associations confounded by diet, bowel-management regimens, antibiotic and other medication use, mobility and physical activity, metabolic status and time since injury. These variables may account for part of the reported association between injury level and microbiota composition (12). Injury level has been reported as an independent determinant of dysbiosis in people with chronic SCI, with individuals with tetraplegia showing greater deviation from reference profiles than those with paraplegia (12); the extent to which this reflects lesion-dependent autonomic dysregulation rather than the confounders listed above remains unresolved, and we therefore report the association without attributing it to a specific mechanism. Small intestinal bacterial overgrowth is also more prevalent in the subacute phase, particularly in tetraplegia, and contributes to gastrointestinal symptoms and to the burden of bowel management (13). Taken together, these data establish that the gut microbiota is altered after SCI, and that the alterations correlate with bowel dysfunction, injury level and circulating inflammatory markers. They do not establish that dysbiosis causes impaired motor recovery. As developed in Sections 5 and 6, most human evidence is associative, and the causal evidence derives almost entirely from rodent models.

Pre-clinical studies have begun to test whether microbiota-targeted interventions can promote neural repair and functional restoration after SCI. These interventions are mechanistically distinct and must be appraised separately: probiotics and prebiotics alter the composition and metabolic output of the resident community; fecal microbiota transplantation (FMT) transfers a whole donor community; antibiotics and bacteriophages deplete or selectively remove taxa; and direct metabolite supplementation, for example with sodium butyrate, bypasses the microbiota entirely. Reported effects in rodent SCI models include reduced lesion-site inflammation after acute-phase antibiotic administration, improved locomotor scores after FMT from uninjured donors, and attenuated microglial activation after butyrate treatment (14–16). These effects are not uniform, however. For the same commercial probiotic preparation, benefit has been reported in a mouse thoracic contusion model and absent in a rat cervical contusion model (10, 17); for several interventions the published results are inconsistent in both direction and magnitude; and the effect on motor outcome is usually a secondary rather than a primary endpoint. We return to these discrepancies in Sections 4 and 6 and in Table 1. Despite the volume of pre-clinical work, clinical translation remains challenging because of the complexity of the gut microbiome, inter-individual variability, and the multifactorial nature of SCI pathology (18). Furthermore, most existing reviews and clinical studies have focused on secondary complications such as bowel dysfunction, neuropathic pain or mood disturbance, with limited attention to motor recovery as a primary outcome. Given that motor restoration is among the foremost concerns of people living with SCI (7), there is a need to consolidate the mechanistic rationale, the pre-clinical evidence and the translational obstacles specifically for microbiota-targeted strategies aimed at motor rehabilitation.

**Table 1:** Pre-clinical studies of microbiota-targeted interventions in spinal cord injury models, with design features and level of evidence.

Intervention	Model	Comparator	Reported effect on motor outcome	Design features reported/not reported	Evidence label	Reference
VSL#3 (multi-strain probiotic)	Mouse, thoracic contusion	Vehicle	Improved locomotor recovery; increased regulatory T cells at the lesion	Sample size, blinding and time points not consistently reported; effect not reproduced in a second model	[SCI-EXP] [INCONSISTENT]	(10)
VSL#3 (multi-strain probiotic)	Rat, cervical contusion	Vehicle	No improvement in locomotor outcome	Explicit negative result; species, injury level, dose and timing differed from the study above	[SCI-EXP]	(17)
FMT from uninjured donors	Mouse, thoracic contusion	Sham or vehicle	Improved locomotor scores; reduced grid-walking errors; axonal preservation and reduced glial scarring	Direction reproduced in independent laboratories; donor-strain engraftment and M1/M2 phenotype definitions variably reported	[SCI-EXP] [CONSISTENT]	(15, 16, 38)
FMT after injury	Rat, cervical contusion	Sham-treated	No significant effect on locomotion; anxiety-like behavior improved	Motor outcome was not the primary endpoint; explicitly negative for locomotion	[SCI-EXP]	(39)
Prebiotics (fructo-oligosaccharides)	No dedicated SCI study identified	-	Not established; no prebiotic intervention study in an SCI model reporting locomotor outcome was identified	Evidence limited to fiber-fermentation and butyrate-producer studies outside SCI	[EXT]	(33) (mechanism only)
Antibiotics (short course, 3–5 days)	Rat and mouse SCI	Untreated or vehicle	Reduced acute inflammatory cell infiltration at the lesion; chronic-phase benefit not established	Microbiota-independent antibiotic effects not excluded; motor outcome rarely a primary endpoint	[SCI-EXP]	(14) (and see text)
Sodium butyrate/SCFA supplementation	Mouse, thoracic contusion	Vehicle	Improved locomotor scores; reduced lesion-site neuroinflammation	Supra-physiological dosing relative to colonic fermentation; mediation by endogenous production untested	[SCI-EXP]	(15, 45)
Bacteriophage cocktail	*Ex vivo* human colon fermentation model (no SCI model)	Control fermentation	Not assessed; community composition and metabolic output only	No SCI model available; strategy unexplored for motor recovery after SCI	[*IN VITRO*] [HYP]	(44)

NR, not reported in the cited source in a form that permits independent verification.

This review has four aims. First, we define the gut-spinal cord axis and distinguish it from the gut-brain axis (Section 2.2). Second, we describe how the gut microbiota changes after SCI and evaluate each proposed mechanistic pathway, explicitly separating evidence generated in SCI from evidence extrapolated from other diseases and models (Sections 3 and 5). Third, we appraise microbiota-targeted interventions in animal models and in people with SCI, and evaluate the interaction between rehabilitation and the microbiota (Sections 4 and 6). Fourth, we analyze the translational barriers_individual variability, timing and duration, safety, and the pre-clinical-to-clinical pipeline-and propose the study designs and reporting standards that would be needed to move the field forward (Sections 7 and 8). Because this is a narrative rather than a systematic review, the search strategy and the evidence-grading convention used throughout are stated explicitly in Section 2, and every major claim is labeled accordingly. Sections 3 and 4 describe the phenomena; Sections 5 and 6 are critical appraisals of the mechanistic and interventional evidence and are intended to be read as graded assessments rather than as further description.

## 2 Review methodology, scope and evidence framework

### 2.1 Search strategy and selection of evidence

This is a narrative review, not a systematic review, and the language used throughout reflects that distinction. We searched PubMed/MEDLINE, Web of Science and Scopus from database inception to July 2026, combining terms for the population (“spinal cord injury” OR “spinal cord injuries” OR “tetraplegia” OR “paraplegia”) with terms for the exposure and the interventions (“gut microbiota” OR “microbiome” OR “dysbiosis” OR “probiotic^*^” OR “prebiotic^*^” OR “fecal microbiota transplantation” OR “FMT” OR “short-chain fatty acid^*^” OR “butyrate” OR “bile acid^*^” OR “intestinal permeability” OR “bacterial translocation” OR “gut-brain axis”). No language restriction was applied other than the authors' ability to translate. Reference lists of the retrieved reviews and of the primary studies identified were screened for additional records.

Records were prioritized in three tiers: primary studies performed in animal models of SCI or in people with SCI; mechanistic studies in other diseases, models or cell systems, included only where they were needed to explain a proposed pathway; and reviews, used for background. Two authors (Z.C. and Z.G.) screened records for relevance and extracted the intervention, model or population, comparator, sample size, timing of assessment and outcome measures where these were reported; disagreements were resolved by discussion with the corresponding author. We did not apply a formal risk-of-bias instrument and we did not perform a quantitative synthesis; where a parameter was not reported in the primary source we say so rather than imputing a value. Consequently, the absence of a study from this review should not be interpreted as evidence of its absence from the literature, and the review should be read as an interpretive synthesis rather than as an exhaustive or reproducible systematic search.

### 2.2 The gut-spinal cord axis and its distinction from the gut-brain axis

Terminology in this field is inconsistent, and precision matters because findings obtained in gut-brain axis research are frequently transferred to SCI without justification. We use gut-spinal cord axis to denote the set of routes through which the gut lumen, its microbial community and their products can influence the spinal cord, and through which spinal cord dysfunction can in turn alter gut function. Three route classes are considered: immune routes, including gut-associated lymphoid tissue (GALT), mesenteric lymph nodes, circulating cytokines and immune-cell trafficking; neural routes, including vagal afferents, the enteric nervous system and-of particular importance in SCI-the sympathetic and sacral parasympathetic control of gut motility, secretion and sphincter function; and humoral routes, dominated by microbially produced metabolites such as SCFAs, secondary bile acids, tryptophan derivatives and lactate (8).

The gut-brain axis shares the first two route classes, but differs from the gut-spinal cord axis in one clinically decisive respect: in SCI, damage to the spinal cord itself interrupts the descending autonomic control of the gut. The axis therefore contains a structural lesion that has no counterpart in brain-directed models. Two consequences follow. First, the effect of the injured spinal cord on the gut is likely to be larger, more direct and better documented than the reverse effect, and the evidence for the two directions should not be treated symmetrically. Second, vagal signaling after SCI operates within a reorganized autonomic system rather than as an intact conduit, so findings from vagotomy or vagal stimulation studies in sepsis, colitis or endotoxaemia cannot be assumed to apply. Comparative evidence from other central nervous system conditions, such as stroke, illustrates both the conceptual appeal and the specificity of these mechanisms (19) [EXT]. We return to both points in Section 5.3.

### 2.3 Evidence framework and how evidence is labeled in this review

Claims about the gut-spinal cord axis are unusually prone to a specific error: a mechanism demonstrated in another disease, or in a germ-free or antibiotic-treated animal without SCI, is presented as established in SCI. To make the provenance of every substantive claim explicit, each major statement in Sections 3–8 carries one or more labels from the scheme in Box 1. We use this device for three reasons: it shows the reader immediately where the evidence is strong and where it is not; it makes explicit which statements are mechanistic hypotheses rather than findings; and it makes the review auditable, so that future authors can update individual claims as new SCI-specific evidence appears.

Box 1 **Evidence labels used throughout this review.**

**Table:** 

**Label**	**Definition, and what the label does not establish**
[SCI-EXP]	Evidence from experimental SCI models (rodent, porcine or other non-human). This is the strongest mechanistic tier available in this field. It does not establish efficacy or safety in humans.
[SCI-CLIN]	Evidence from people with SCI, whether observational or interventional. Observational data establish association only; they do not establish causation, because diet, medication, bowel management, activity level and time since injury differ systematically between groups.
[EXT]	Evidence from experimental or clinical settings other than SCI (for example gut-brain axis studies, multiple sclerosis, stroke, inflammatory bowel disease, irritable bowel syndrome, sepsis, oncology, critical care). It supports plausibility; it is not transferable to SCI without verification.
[*IN VITRO*]	Cell culture or *ex vivo* systems, including organoids, co-culture platforms and fecal fermentation models. Useful for mechanism; no inference about organ-level or functional outcomes.
[HYP]	An explicitly hypothetical mechanistic sequence, proposed by the authors or by the cited source, that has not been empirically demonstrated in SCI. The label is applied to whole pathways and to individual mediating steps.
[CONSISTENT]	The direction of the effect has been reproduced by two or more independent SCI studies.
[SINGLE]	Reported in a single SCI study and not yet independently replicated. Single-study findings are reported as such.
[INCONSISTENT]	SCI studies of the same or comparable intervention report opposing results in functional outcome.
No label	Definitional statements, general background supported by reviews, or statements explicitly identified in the text as the authors' appraisal. Absence of a label does not imply strong evidence.

Two further conventions apply. Where the same claim is supported by studies that disagree, we report both results rather than selecting one, and we state the design features that differ (species, injury level and model, dose, route, timing, and outcome measure). Where a statement is our own inference rather than a published finding it is marked [HYP] or stated as such in the text. A label reflects the strength of the evidence available at the time of writing, not the plausibility of the mechanism.

## 3 Gut dysbiosis after spinal cord injury: compositional patterns and pathophysiological consequences

### 3.1 Compositional changes and their temporal and spatial pattern

Compositional change after SCI is well documented, but its temporal resolution is uneven, and one frequently repeated formulation-that dysbiosis is “observable within hours post-injury”-is not supported by the primary literature. In rodent SCI models, compositional differences relative to uninjured controls are detectable within the first days after injury and become progressively more pronounced over weeks, with the largest effects reported after high-thoracic and cervical injuries (10, 20, 21) [SCI-EXP]. Sampling at sub-daily resolution has not been reported in any species, so unless future studies demonstrate otherwise the appropriate statement is that dysbiosis emerges over days, not hours. In humans, the earliest available sampling windows are admission and the early acute phase; at these time points community composition already clusters by injury severity, and pre-injury baseline samples are almost never available, which limits inference about the direction of change (11, 22) [SCI-CLIN].

Early changes described in humans include lower alpha-diversity (Shannon and related indices), indicating reduced richness and evenness, together with altered relative abundance of several genera (22) [SCI-CLIN]. Phylum-level imbalance is usually summarized as a change in the Firmicutes-to-Bacteroidetes (F/B) ratio, but we deliberately de-emphasize this metric. The F/B ratio is a coarse, composition-dependent index that is highly sensitive to methodological choices (16S hypervariable region, primer set, sequencing platform, normalization), varies widely among healthy individuals, and has not been shown to predict any clinical or functional outcome after SCI; its use as a stand-alone marker of dysbiosis has been criticized (23). Where phylum-level shifts are reported after SCI we therefore present them alongside metrics that are more informative for mechanism and more closely anchored to function: alpha-diversity and evenness, composition at genus and species level, community functional potential (for example the abundance of butyrate-synthesis gene clusters), and measured metabolite output such as fecal SCFA concentration.

Longitudinal studies show that gut microbiota composition and neuroinflammatory markers change over a similar post-injury time course (22). This co-variation is consistent with, but does not demonstrate, a causal link, and the proposition that microbial signatures could serve as biomarkers of disease progression or of therapeutic response remains a hypothesis: no prospective study in SCI has shown that a baseline microbial signature predicts the clinical course or the response to a microbiota-targeted intervention. We therefore present this application as a testable proposal requiring prospective validation rather than as an established capability [HYP].

The mechanistic loop proposed for this section-SCI-induced autonomic dysfunction impairs intestinal motility and secretion, which favors overgrowth of pathogenic taxa and loss of commensals, which in turn promotes systemic inflammation and may hinder neurological recovery_is only partly supported by direct evidence. Experimentally demonstrated in SCI: reduced colonic transit and altered motility after high-thoracic injury, segment-dependent changes in epithelial permeability and mesenteric perfusion, and altered luminal community composition (10, 14, 24) [SCI-EXP]. Not demonstrated: that the microbial change itself, rather than altered motility, medication exposure or dietary change, drives the systemic inflammatory change; and not demonstrated in humans at all: that any component of the loop causally limits neurological recovery [HYP]. We therefore treat this loop as a plausible, partially supported working model. Early microbiota-targeted intervention is a rational consequence of the model, not a validated strategy, and it should not be described as a demonstrated mechanism. Figure 1 summarizes this working model of the gut-spinal cord axis after spinal cord injury.

**Figure 1:**
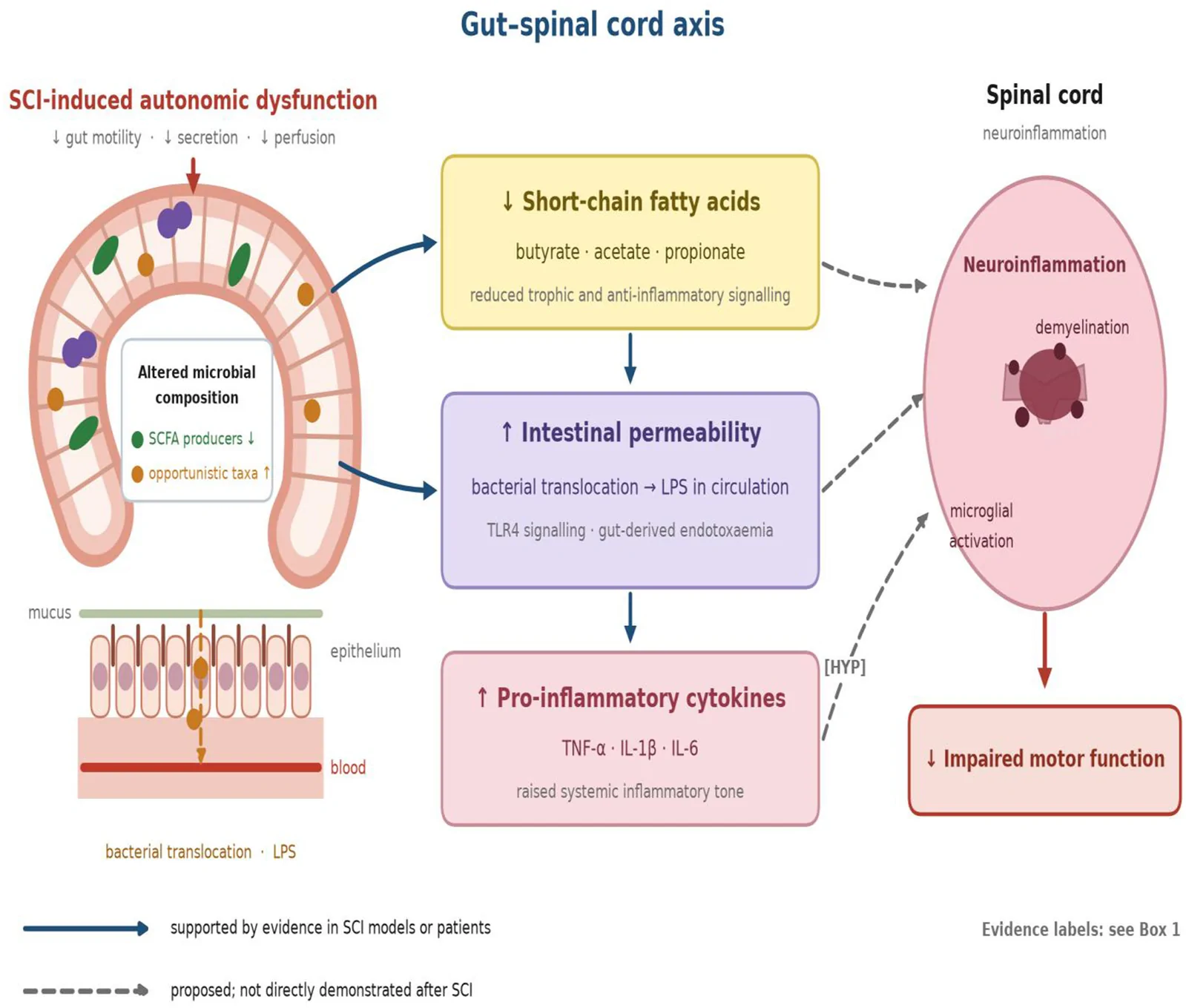
The gut-spinal cord axis after spinal cord injury. Injury-induced autonomic dysfunction alters intestinal motility, secretion and perfusion; these changes are accompanied by altered microbial composition, reduced short-chain fatty acid production and increased intestinal permeability, which together with a raised systemic inflammatory tone may aggravate neuroinflammation and limit motor recovery. Solid arrows denote relationships supported by evidence obtained in SCI models or in patients with SCI; dashed arrows denote proposed relationships that have not been directly demonstrated after SCI. Evidence labels are defined in Box 1.

### 3.2 Microbial metabolites: a conceptual overview

Gut microbial metabolites are the principal molecular interface between the gut lumen and the central nervous system, and the short-chain fatty acids (SCFAs) butyrate, propionate and acetate are the best characterized. They are produced by bacterial fermentation of dietary fiber and act on host cells through at least three routes: activation of G protein-coupled receptors (GPR41/FFAR3, GPR43/FFAR2, GPR109A), inhibition of class I histone deacetylases (HDACs), and, for butyrate, use as an energy substrate by colonocytes (8). Secondary bile acids are a second major class: deoxycholic acid and lithocholic acid are generated by microbial 7alpha-dehydroxylation of primary bile acids and signal through the farnesoid X receptor (FXR) and the Takeda G protein-coupled receptor 5 (TGR5) (25). Tryptophan metabolism by gut bacteria generates indole derivatives, kynurenine and serotonin (5-HT), some of which act through the aryl hydrocarbon receptor (AHR) (26, 27). Lactate, succinate and trimethylamine derivatives complete the current catalog of candidate mediators. Table 2 summarizes these metabolites, their principal microbial sources, their proposed mechanisms, and the level of evidence available in SCI. Because these pathways are conceptually attractive and are well supported in other diseases, there is a strong temptation to assume that they operate after SCI. They may do so, but the evidence is uneven, and Section 5.1 grades it pathway by pathway. Two considerations are worth stating at the outset. First, many molecular effects of SCFAs have been characterized in cultured microglia, astrocytes or intestinal epithelial cells, or in models of colitis, multiple sclerosis, stroke or sepsis; such studies establish plausibility, not occurrence after SCI [EXT] [*IN VITRO*]. Second, the concentrations achieved by systemic administration of a metabolite such as sodium butyrate generally exceed those produced by colonic fermentation by orders of magnitude, so a benefit from pharmacological dosing does not by itself demonstrate that endogenous microbial production achieves the same effect. This distinction is frequently blurred in the literature and is one reason why studies of pharmacological HDAC inhibitors are not interchangeable with studies of microbial metabolites. Figure 2 summarizes the proposed pathway classes through which the gut microbiota and its products may influence recovery after spinal cord injury; these are appraised individually in Section 5.

**Table 2:** Gut microbiota-derived metabolites proposed to influence motor recovery after spinal cord injury, with the level of evidence currently available in SCI.

Metabolite	Principal microbial sources	Proposed mechanism	Reported effect on motor or functional outcome	Evidence available in SCI
Butyrate	Firmicutes: *Clostridium* clusters IV/XIVa, *Faecalibacterium, Butyricimonas*	HDAC inhibition; GPR109A activation; upregulation of BDNF and other neurotrophic factors; anti-inflammatory microglial polarization	Improved locomotor scores and reduced neuroinflammation after exogenous administration in rodent SCI models	[SCI-EXP] for exogenous administration; [*IN VITRO*] for the HDAC mechanism in microglia
Propionate	Bacteroidetes; Negativicutes (*Veillonella*)	GPR43/FFAR2 activation; Treg induction; suppression of Th17 responses	Reduced neuroinflammation and restored Treg/Th17 balance in a rodent SCI model after SCFA administration	[SCI-EXP]
Acetate	*Bifidobacterium; Akkermansia muciniphila; Prevotella* (species-dependent)	GPR41/FFAR3 and GPR43 signaling; microglial modulation; histone acetylation	Attenuated microglial activation and neuroprotection, demonstrated largely in non-SCI models	[EXT]; not established in SCI
Secondary bile acids (deoxycholic acid, lithocholic acid)	*Clostridium*; *Lactobacillus*; *Bifidobacterium* (7alpha-dehydroxylation of primary bile acids)	FXR and TGR5 activation; NF-kappaB suppression; anti-apoptotic signaling; modulation of colonic motility and secretion	Myelin debris clearance and remyelination have been proposed but not demonstrated in SCI. Effects on colonic motility and secretion are relevant to neurogenic bowel dysfunction	[HYP] for remyelination in SCI; [EXT] for motility and barrier effects
Tryptophan metabolites (indole derivatives, kynurenine)	*Bifidobacterium*; *Lactobacillus*; *Clostridium sporogenes*	Aryl hydrocarbon receptor activation; IL-22 production; intestinal barrier maintenance; kynurenine pathway modulation	Barrier protection and indirect reduction of systemic inflammation; effects characterized mainly outside SCI. One SCI study reported reduced neuroinflammation after *Limosilactobacillus reuteri* administration via tryptophan metabolism	[EXT]; [SCI-EXP] for the single SCI study
Serotonin (5-HT)	Host enterochromaffin cells, modulated by spore-forming gut bacteria	Peripheral 5-HT regulates gut motility and vascular tone and is largely sequestered by platelets; it does not cross the blood-brain barrier appreciably	Lumbar 5-HT release correlates with locomotor recovery in SCI, but this is an intrinsic spinal serotonergic mechanism rather than a microbial one	[SCI-EXP] for the spinal mechanism; [HYP] for a microbial contribution
Lactate	*Lactobacillus*; *Bifidobacterium*; *Enterococcus*	GPR81 signaling; alternative energy substrate for neurons; promotion of angiogenesis	Metabolic support for regenerating neural tissue; not examined in SCI as an outcome-directed intervention	[EXT]; not established in SCI
Succinate	*Bacteroides; Prevotella; Phascolarctobacterium*	GPR91/SUCNR1 signaling; pro-inflammatory macrophage and Th17 responses	Proposed pro-inflammatory contribution, documented in non-SCI systems and not in SCI	[EXT]; [HYP] in SCI

Labels follow Box 1. “Not established in SCI” indicates that the mechanism has not been demonstrated in an SCI model; it does not imply that the mechanism is absent.

**Figure 2:**
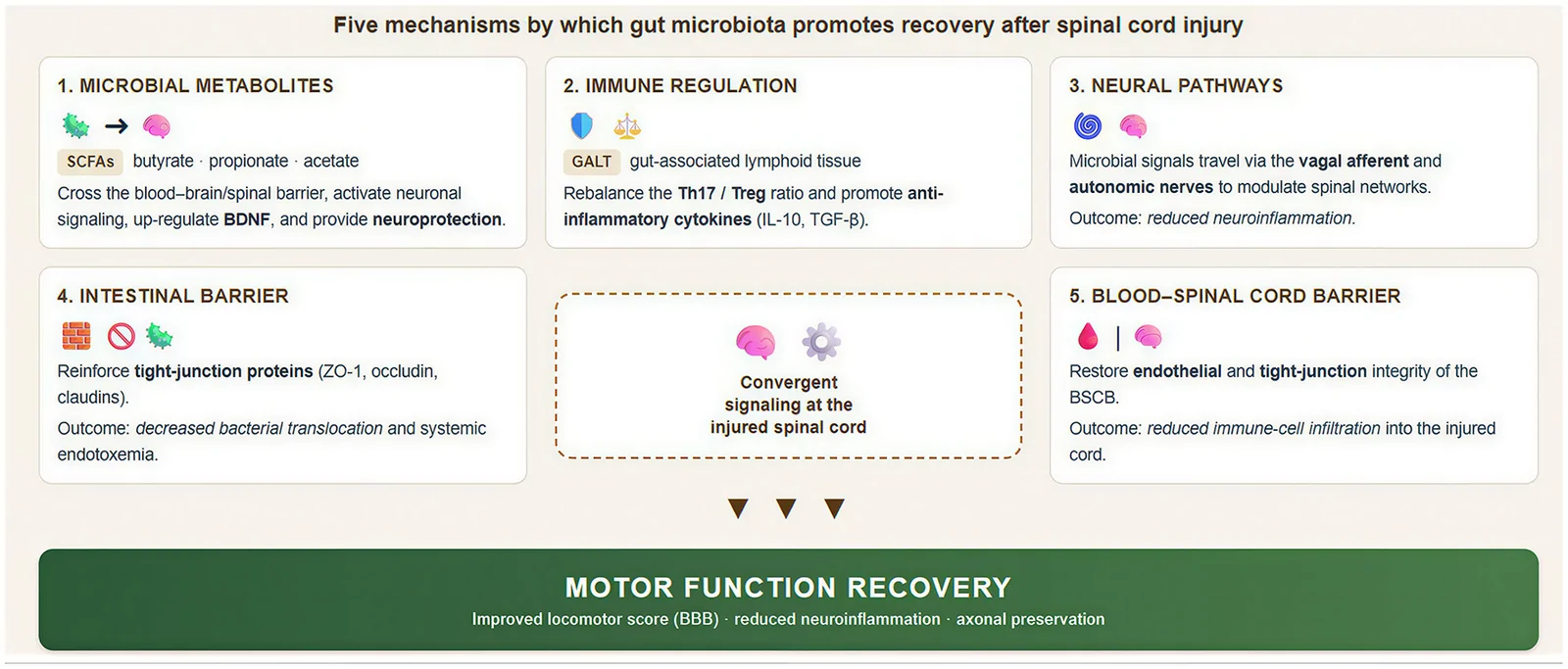
Proposed mechanistic pathways linking the gut microbiota to motor recovery after spinal cord injury. Five pathway classes are shown: microbial metabolite signaling (short-chain fatty acids, bile acids, tryptophan derivatives), immune regulation (gut-associated lymphoid tissue, mesenteric lymph nodes, Treg/Th17 balance, dendritic cells), neural signaling (vagal afferents, autonomic outflow, enteric nervous system), the intestinal barrier, and the blood-spinal cord barrier. Pathways shown in gray are supported principally by evidence from diseases other than SCI and are presented as hypotheses rather than as established mechanisms; see Sections 5.1–5.5 and Box 1.

### 3.3 Intestinal barrier disruption and systemic inflammation

SCI-induced autonomic dysfunction alters gut physiology at several levels: intestinal blood flow falls, mucus and secretory output change, and the expression of tight junction proteins such as occludin, claudin-1 and ZO-1 is reduced, with the result that epithelial permeability increases (24, 28) [SCI-EXP] [SCI-CLIN]. “Leaky gut” is best defined operationally rather than as an independent pathological entity, and it should be quantified. Accepted approaches include urinary recovery of orally administered probes (lactulose/mannitol ratio, 51Cr-EDTA), FITC-dextran flux, *ex vivo* Using chamber measurement of transepithelial electrical resistance, and quantification of tight-junction proteins by immunoblot or immunofluorescence (24, 29). The choice of assay matters, because the different methods measure different aspects of permeability and do not agree closely with one another; in the SCI literature the method used is often not reported, which limits comparability between studies. Whether increased permeability is accompanied by translocation of bacteria, or only of bacterial products, is a separate question that the current literature rarely addresses. Increased permeability, elevated circulating endotoxin and systemic inflammation are frequently presented as a single causal chain, but each link requires its own evidence. Circulating lipopolysaccharide (LPS) is measured with assays of variable specificity, and plasma endotoxin can also have non-gut sources, since LPS is absorbed with dietary fat and transported on chylomicrons; demonstration of translocation requires detection of viable bacteria or of microbial nucleic acid in blood, mesenteric lymph nodes or extraintestinal organs, ideally with strain-level confirmation, and translocation should not be inferred from recovery of diversity indices (29, 30). We therefore present the sequence “barrier failure to bacterial translocation to exacerbation of spinal cord inflammation and neuronal apoptosis” as a mechanistic hypothesis that has not been demonstrated in SCI [HYP], while noting that the association between barrier dysfunction and systemic inflammation is reasonably well supported [SCI-EXP] [SCI-CLIN]. A related claim that is frequently repeated requires correction. It states that restoring barrier integrity with *Lactobacillus* species “significantly reduces serum inflammatory cytokine levels and improves motor function scores”. The supporting citation was a study of visceral hypersensitivity in irritable bowel syndrome (IBS) and not an SCI study (31) [EXT]. In rodent SCI models, several *Lactobacillus*-based preparations have been reported to reduce circulating and lesion-site pro-inflammatory mediators and to improve locomotor scores, but the effect on motor outcome varies between preparations and between models, and the causal link to barrier restoration has not been tested (Section 4.1 and Table 1).

## 4 Microbiota-targeted interventions in animal models of spinal cord injury

Figure 3 summarizes the translational pathway from pre-clinical findings to clinical practice for microbiota-targeted therapies and identifies the evidence gap between the two.

**Figure 3:**
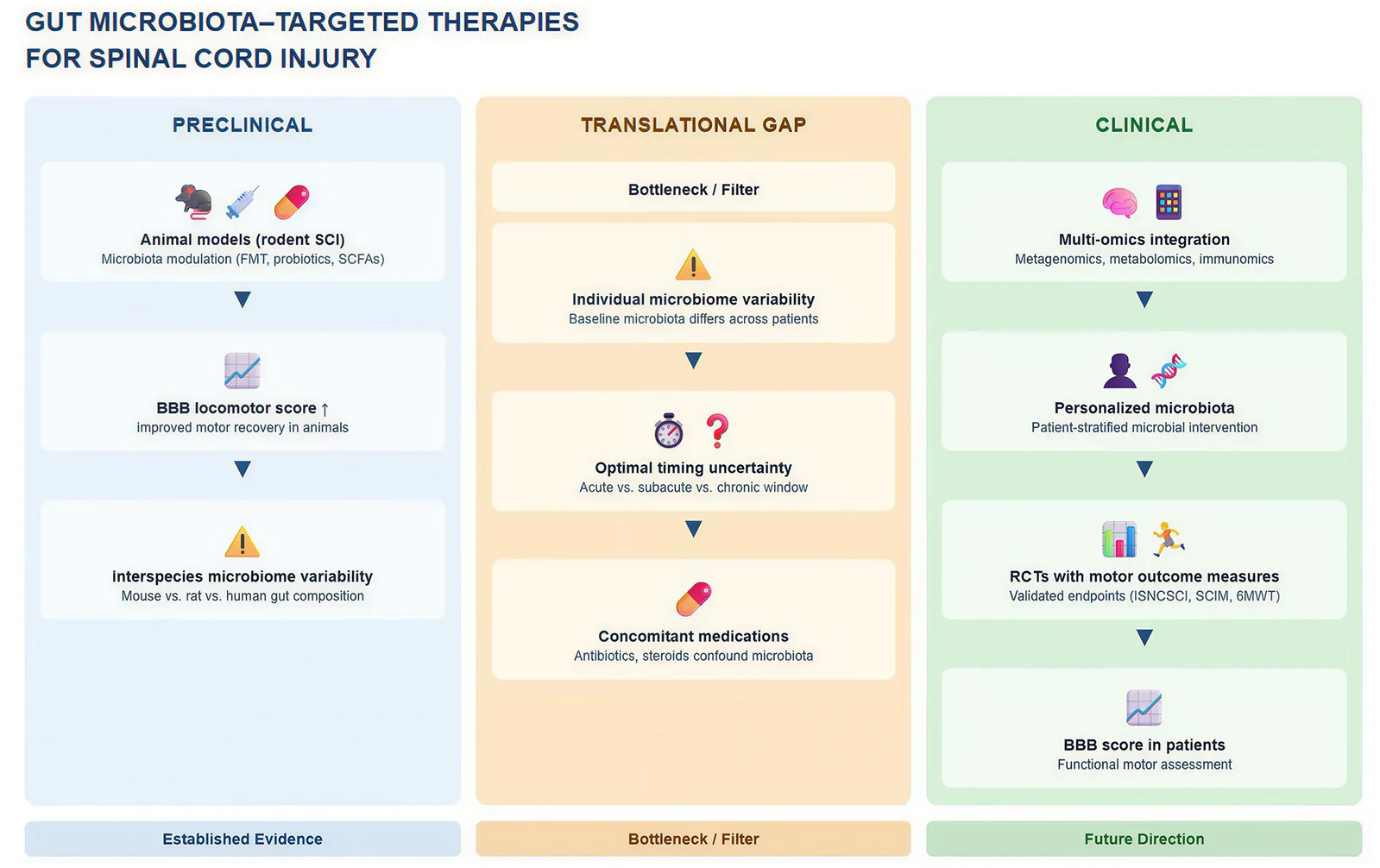
Translational pathway for microbiota-targeted therapies in spinal cord injury, showing the evidence gap that separates pre-clinical findings from clinical practice. Pre-clinical studies establish feasibility and mechanistic plausibility; demonstration of causal mediation in SCI models is largely missing; human evidence consists of observational associations and interventional studies without motor endpoints; and randomized trials with validated motor outcomes remain to be performed. Standardized reporting, harmonized intervention protocols and predefined safety monitoring are prerequisites at every stage.

### 4.1 Probiotics and prebiotics

The pre-clinical literature on probiotics in SCI is smaller and less consistent than its frequency of citation suggests, and two studies using the same commercial preparation illustrate why the field needs careful reading. In a mouse thoracic contusion model, oral administration of the multi-strain preparation VSL#3 after injury increased the number of regulatory T cells at the lesion site and improved locomotor recovery relative to vehicle-treated animals (10) [SCI-EXP]. In a rat model of cervical contusion, the same preparation given according to the manufacturer's instructions did not improve locomotor outcomes, and the authors explicitly reported an absence of benefit (17) [SCI-EXP] [INCONSISTENT]. Because species, injury level, dose, bacterial viability and timing of administration all differed, the discrepancy cannot be attributed to a single variable; it does demonstrate that species-level and model-level generalization is unsafe, and that a single positive result in one model should not be described as an established effect. Claims about the magnitude of benefit in this literature also require qualification. It is frequently claimed that probiotics produce an increase of 2–3 points on the Basso, Beattie and Bresnahan (BBB) scale in approximately 60% of treated animals. These figures cannot be verified in the primary sources cited, which do not report the effect in this form, and we do not reproduce them here. Where BBB or Basso Mouse Scale improvements are reported, we now state the model, the comparator, the time point and whether the difference was statistically significant, and we indicate explicitly when the primary source does not report these details (Table 1).

Mechanistically, several *Lactobacillus* strains reduce pro-inflammatory signaling in microglia, and strain-dependent differences are well documented: in lipopolysaccharide-exposed animals, *Lacticaseibacillus rhamnosus* GG and *Limosilactobacillus reuteri* differed in their effects on microglial polarization and on gut barrier function (32). That study did not use an SCI model and did not measure locomotor recovery, so it supports the general proposition that effects are strain-specific rather than the specific claim that one strain is superior for motor recovery after SCI [EXT]. The same caution applies to prebiotics. Fructo-oligosaccharides selectively promote Bifidobacterium and increase butyrate availability, and fructo-oligosaccharide utilization by butyrate-producing commensals is well characterized (33); however, we identified no dedicated prebiotic intervention study in an SCI model that measured locomotor outcome, and the downstream sequence proposed for SCI-increased butyrate, increased spinal brain-derived neurotrophic factor (BDNF), axonal regeneration, improved function_has not been demonstrated end to end within an SCI study [HYP]. The neurotrophic-factor component of that sequence is, separately, well supported: neurotrophin-3 delivery promotes axonal regeneration and partial functional recovery after experimental SCI (34) [SCI-EXP], but that evidence concerns the neurotrophin itself rather than the microbial pathway proposed to increase it. A claim about treatment duration also requires revision. It is often stated that “sustained treatment over several weeks yields more pronounced benefits”, but no dose-duration comparison in SCI has been published, and the studies cited differed in model, preparation and outcome measurement as well as in duration. Duration of treatment is therefore not a demonstrated determinant of efficacy and should be treated as an unresolved design question (Section 7.2).

In summary, probiotics and prebiotics are the most studied microbiota-directed interventions in SCI, but the evidence base consists of a small number of studies that disagree in direction, use different species, strains, doses and models, and rarely report motor outcome as the primary endpoint with adequate statistical detail. Strain screening and characterization have been formalized in other contexts (35–37), and those methods are transferable, but they do not by themselves establish efficacy. The appropriate conclusion is that this is a promising but unconsolidated area of pre-clinical work, not that a robust pre-clinical foundation for probiotic therapy in SCI already exists.

### 4.2 Fecal microbiota transplantation

FMT is the intervention for which the largest and most consistent pre-clinical effects on motor function have been reported in SCI models. In mice with thoracic contusion, transplantation of fecal material from uninjured donors reduced lesion-site neuroinflammation and improved locomotor scores, and independent studies have reported improved motor performance together with reduced grid-walking errors and evidence of axonal preservation and reduced glial scarring (15, 16, 38, 39) [SCI-EXP] [CONSISTENT]. Because these reports come from more than one laboratory, the direction of the functional effect is more reproducible than for probiotics. The mechanistic interpretation, however, is weaker than the descriptive result. Restoration of alpha-diversity to values comparable to those of uninjured controls is frequently described as “successful engraftment and reconstitution of a balanced microbial community”, but recovery of a diversity index does not demonstrate establishment of donor strains, because diversity can increase without donor organisms persisting. In human FMT for recurrent Clostridioides difficile infection, strain-resolved tracking has shown that engraftment is highly variable and depends on donor-recipient strain competition and on the recipient's existing community rather than on simple recovery of diversity (30) [EXT]. The same distinction should be drawn in SCI, where strain-level data are currently absent: engraftment should be demonstrated by donor-strain tracking or by donor-specific marker organisms and reported separately from diversity restoration.

The two quantitative immunological and behavioral claims most often cited also require revision. They state an approximately 40% increase in M2-type macrophages and a 30% reduction in grid-walking errors; neither figure can be verified as stated in the cited sources, which do not report the effect in this form, and both omit the information needed to judge an effect_how M1 and M2 phenotypes were defined and quantified, the sample size per group, the sampling site, the time point, and whether the difference was statistically significant. We have therefore removed the percentages and now describe the direction of the reported effects. We also note that a reduction in grid-walking errors indicates improved sensorimotor integration and is not specific to voluntary motor control, so it cannot be equated with locomotor recovery as assessed by the BBB scale. This is not merely a presentational point: a future systematic review of this literature would be unable to pool the available results, because the outcome definitions and reporting are not comparable.

Whether the functional benefit of FMT is mediated by SCFAs produced by the restored community is a hypothesis rather than a demonstrated mechanism. The sequence “FMT to increased SCFA availability to oligodendrocyte precursor cell differentiation and remyelination to improved motor performance” has not been demonstrated as a whole in any SCI model. The component linking SCFAs to remyelination derives largely from models of multiple sclerosis and from cell systems (40) [EXT], and although SCFA-producing taxa and SCFA concentrations do change after FMT in some SCI studies (15), the mediating step has not been tested by SCFA depletion, receptor antagonism or supplementation-controlled designs. We therefore present the mechanism as [HYP]. The safety discussion in this literature also generalizes findings from FMT in other indications to SCI. Adverse metabolic and immunological outcomes have been described after FMT in non-SCI populations, and the largest long-term safety datasets come from recurrent C. difficile infection (41, 42) [EXT]; in SCI specifically, no long-term safety data have been reported. “Healthy donor” likewise requires definition rather than assumption: donor eligibility is determined by a combination of clinical history, serological and microbiological screening, body-mass index and metabolic criteria, medication exposure, diet and travel history, and the criteria used in SCI trials should be stated explicitly (41). Comparable gut-immune interactions have been described in other disease contexts, for example in acute liver injury models, where FMT altered the Treg/Th17 balance (43) [EXT]; such studies are informative about mechanism but do not establish what happens after SCI.

Accordingly, the frequently repeated summary statement that FMT “enhances motor recovery after SCI by restoring gut microbial homeostasis and promoting anti-inflammatory macrophage polarization” is not supportable as written. The defensible statement is narrower: FMT has been reported to change the gut community and the local immune environment after SCI and to be accompanied by improved locomotor outcomes in several animal models, but the causal contribution of the microbial or immunological change to the functional improvement has not been demonstrated, and no clinical trial in people with SCI has yet tested the intervention with a motor endpoint [SCI-EXP] [HYP].

### 4.3 Antibiotic-mediated microbiota manipulation, bacteriophages and direct metabolite supplementation

Antibiotics occupy a different position from probiotics and FMT, because they can act on the host independently of the microbiota. The beneficial and detrimental consequences of antibiotic administration are usually attributed almost entirely to microbiota depletion. This attribution is not warranted. Aminoglycosides, beta-lactams, fluoroquinolones and macrolides have immunomodulatory and neuroactive effects of their own, and alter mitochondrial function and host signaling in ways that do not require bacteria; conversely, antibiotics also alter host physiology indirectly through the loss of colonization resistance. Attributing an observed change in neuroinflammation or recovery to the microbiota therefore requires a design that isolates the microbial contribution, for example comparison with germ-free or gnotobiotic animals, microbiota transfer into antibiotic-treated hosts, or selective depletion using non-absorbable agents. Most available SCI studies do not include such controls, and the mechanism therefore remains ambiguous [SCI-EXP] [HYP]. With that caveat, the reported pattern in SCI models is that early, short-course antibiotic treatment reduces acute inflammatory cell infiltration at the lesion site, whereas prolonged depletion is associated with impaired repair in the chronic phase, consistent with a role for the commensal community in supporting recovery (14) [SCI-EXP]. The commonly repeated statement that prolonged antibiotic exposure causes “irreversible” loss of beneficial populations should be qualified: the magnitude and reversibility of the change depend on the antibiotic, its spectrum and duration, host factors, and the availability of a reservoir for recolonization, and recovery is often partial and slow rather than absent. Accordingly, we refer to the antibiotic- and host-dependent extent of recovery rather than to “irreversible dysbiosis” (Section 7.3).

Bacteriophage therapy and direct metabolite supplementation are sometimes discussed together with probiotics and FMT, but they are mechanistically distinct: phages selectively lyse targeted bacterial strains without removing the surrounding community, whereas metabolite supplementation bypasses the microbiota altogether. Phage cocktails have been shown to alter community composition and metabolic output in *ex vivo* colonic fermentation models (44) [*IN VITRO*], but they have not, to our knowledge, been tested for motor recovery after SCI; bacteriophage therapy is therefore an unexplored strategy rather than a pre-clinical candidate [HYP]. Direct administration of SCFAs supports the general proposition that metabolite availability can influence recovery. In rodent SCI models, butyrate or SCFA supplementation has been reported to reduce microglial activation and lesion-site pro-inflammatory cytokine expression, to increase neurotrophic factor expression, and to improve locomotor scores (15, 45) [SCI-EXP]. However, “SCFA supplementation” and “FMT” are not interchangeable interventions: the former tests the sufficiency of a metabolite, the latter the sufficiency of a community, and they answer different mechanistic questions. Which microbial metabolite reaches the spinal cord, at what concentration, and whether that concentration is physiologically attainable from colonic fermentation, remain largely unresolved; we return to this in Section 5.1 [HYP]. Community-modifying interventions of non-microbial origin have also been studied in SCI models, for example plant-derived polysaccharides that alter the gut community and reduce microglial activation (46, 47) [SCI-EXP]; these are informative about the plausibility of the axis but do not resolve the question of which microbial signal matters.

These findings indicate that antibiotics, probiotics and FMT should not be grouped as interchangeable means of “modulating the microbiota”. Each answers a different question-depletion, supplementation and community replacement, respectively-and their effects on motor recovery differ in direction and magnitude. Table 1 organizes the available pre-clinical evidence by intervention, model, comparator and outcome, and states the level of evidence and the principal limitation of each study. Reading the literature in this structured way makes two features clear: the number of studies per intervention is small, and the number that report motor outcome as a primary, adequately powered endpoint is smaller still.

In summary, microbiota-targeted interventions improve locomotor outcomes in some rodent SCI models, and the most reproducible functional signal comes from FMT. The consistency of the functional finding does not extend to the mechanism: for no intervention has the causal chain from a specific microbial or metabolite change to a specific functional improvement been demonstrated within a single SCI study. This is the central limitation of the pre-clinical literature and the main reason that clinical expectations should be calibrated accordingly (Sections 6 and 7).

## 5 Mechanistic pathways linking the gut microbiota to motor recovery: a graded appraisal

Section 3 introduced the compositional and physiological changes that follow SCI. This section evaluates, pathway by pathway, how far each proposed mechanism is supported by evidence obtained in SCI, using the labels defined in Box 1. It is intended as a critical appraisal rather than a further description of mechanism, and where evidence is transferable only by analogy this is stated explicitly.

### 5.1 Microbial metabolite pathways

#### 5.1.1 Short-chain fatty acids

The molecular actions of SCFAs-HDAC inhibition, GPR41/GPR43/GPR109A signaling, and modulation of histone acetylation-are established in cell systems and in non-SCI disease models [*IN VITRO*] [EXT] (8). In SCI specifically the evidence is narrower. Systemic or local administration of sodium butyrate has been reported to reduce microglial and astrocytic activation and lesion-site pro-inflammatory cytokine expression and to improve locomotor scores in rodent contusion models (45, 48) [SCI-EXP]. The effect of sodium butyrate on microglial polarization has also been characterized *ex vivo* after oxygen-glucose deprivation, a model that contains neither gut nor microbiota [*IN VITRO*] (48). Two qualifications are important: the concentrations used in supplementation studies typically exceed those attainable from colonic fermentation, and the effect of an administered HDAC inhibitor does not establish that endogenous microbial SCFA production produces the same change. This is the point at which an inference that is frequently drawn needs to be separated out. Sodium butyrate is commonly grouped with valproic acid (VPA) as evidence for the therapeutic potential of “targeting epigenetic regulation via microbial metabolites”. VPA is neither a microbial metabolite nor an SCFA; it is a pharmacological HDAC inhibitor. Its protective effect on blood-spinal cord barrier integrity and its attenuation of microgliosis and neuroinflammation after SCI are well documented and are relevant in their own right (49–51) [SCI-EXP], but they constitute pharmacological evidence about HDAC inhibition, not evidence about microbiota-derived metabolites. The two lines of evidence are presented separately throughout this review, because conflating them overstates what is known about the microbial contribution. Receptor-level evidence is also thinner in SCI than the literature implies. GPR43-mediated effects on the Treg/Th17 balance have been demonstrated after SCFA administration in a rodent SCI model (52) [SCI-EXP]; the contributions of GPR41, GPR109A and specific HDAC isoforms to recovery after SCI have not been dissected using receptor- or isoform-specific manipulations within an SCI model [HYP].

#### 5.1.2 Bile acids

Secondary bile acids influence intestinal barrier integrity, immune-cell trafficking and epithelial differentiation through FXR and TGR5 (25, 53) [EXT]. It is often stated that deoxycholic acid “enhances clearance of myelin debris at the injury site and facilitates remyelination and repair” after SCI. We have been unable to locate direct evidence for this in SCI models; the supporting studies had been conducted in intestinal, metabolic or hepatic contexts, and the cited reference addressed intestinal barrier dysfunction under hypoxia rather than the spinal cord (53) [EXT]. Bile-acid-mediated myelin clearance and remyelination after SCI is therefore presented as a hypothesis [HYP]. Bile acids remain highly relevant to SCI in a different respect: as ligands at TGR5 on enteric neurons and enteroendocrine cells they modulate colonic motility and secretion, which is directly pertinent to neurogenic bowel dysfunction, although not to motor recovery (25).

#### 5.1.3 Gut-derived neurotransmitter precursors

A frequently repeated formulation holds that gut-derived gamma-aminobutyric acid (GABA) and serotonin “can cross the blood-brain barrier or act via the vagus nerve, directly affecting central nervous system plasticity and motor neuron function”. This formulation conflates three steps that require separate evidence: microbial production or modulation of the transmitter or its precursor in the lumen; its appearance in the systemic circulation at physiologically meaningful concentrations; and its access to, and functional effect on, spinal cord circuits. Serotonin is the clearest illustration of why the distinction matters: peripheral 5-HT is largely taken up and stored by platelets, acts predominantly on gut motility and vascular tone, and does not cross the blood-brain barrier appreciably [EXT, consensus]. In SCI the well-documented serotonergic phenomenon is intrinsic to the spinal cord rather than microbial in origin: the return of 5-HT immunoreactivity and of stimulus-evoked 5-HT release in the lumbosacral cord correlates with locomotor recovery after incomplete injury (54, 55) [SCI-EXP]. That is a spinal mechanism and should not be attributed to the microbiota. Bacterial production of GABA and of tryptophan-derived AHR ligands is documented and can act on enteric and immune cells (26, 27), but whether these compounds reach spinal circuits in functionally relevant concentrations after SCI is unknown [HYP]. The claim is stated below in that restricted form, and direct effects on spinal plasticity should not be described as established.

#### 5.1.4 Bidirectional regulation: what is and is not supported

The mechanisms most often discussed under this heading are predominantly anti-inflammatory, yet the heading that this literature usually attaches to them-“bidirectional regulation of neuroinflammation by microbial metabolites”-implies regulation in both directions. Bidirectional potential is plausible and is supported in non-SCI systems: polysaccharide A from Bacteroides fragilis is immunosuppressive, whereas lipopolysaccharide, flagellin and the expansion of Enterobacteriaceae signal through TLR4, TLR5 and the NLRP3 inflammasome to amplify inflammation; deoxycholic acid is pro-inflammatory in the colonic epithelium at high concentrations; and succinate promotes pro-inflammatory macrophage responses and Th17 differentiation (29, 56) [EXT]. In SCI, however, we are not aware of a study that has demonstrated a microbially produced metabolite aggravating spinal cord inflammation, and a carefully controlled example from another neurological disease shows that perturbing the microbiota does not necessarily modulate ongoing disease (57) [EXT]. This section is therefore framed as an account of metabolite pathways in general, and we state explicitly that pro-inflammatory potential is extrapolated from other systems rather than demonstrated after SCI [HYP]. Demonstrating it would require a design in which a defined metabolite or defined community is shown to worsen a defined inflammatory or functional outcome in an SCI model. Finally, the statement that strategies increasing beneficial metabolite production “hold promise” for functional recovery has been linked to the underlying evidence. Metabolite-directed interventions have improved locomotor scores in a small number of rodent SCI studies, generally with a single intervention, a single dose and a single outcome assessment, and rarely with a pre-specified primary endpoint (15, 45) [SCI-EXP, limited]. “Promise” is therefore justified as a rationale for testing, not as a summary of demonstrated efficacy.

### 5.2 Immune regulatory pathways

#### 5.2.1 Gut-associated lymphoid tissue and mesenteric lymph nodes

GALT and the mesenteric lymph nodes act as an immunological firewall that limits translocation of luminal bacteria and their products; the anatomy, cellular composition and consequences of failure of this barrier are well characterized, but principally in infection, inflammatory bowel disease and sepsis (58) [EXT]. After SCI, mesenteric perfusion and regional epithelial permeability change in a segment-dependent manner in the rat, indicating that the substrate for a firewall defect exists after injury (24) [SCI-EXP]. Whether these changes are followed by translocation of viable bacteria, and whether translocation then contributes to lesion-site neuroinflammation and impaired recovery, has not been demonstrated directly in SCI, so the causal sequence is presented as a hypothesis [HYP]. Demonstrating it would require detection of translocated organisms rather than of circulating endotoxin, together with an intervention that blocks translocation without altering the community. T cells and the Treg/Th17 balance. A dysregulated balance between regulatory T cells and IL-17-producing cells is a plausible contributor to maladaptive post-injury immunity. In a rodent SCI model, SCFA administration shifted this balance toward Treg cells and away from IL-17+ gamma-delta T cells and was accompanied by improved recovery (52) [SCI-EXP]. This is the most direct evidence in the field linking a defined microbial metabolite, a defined immune population and a functional outcome within the same SCI experiment; even here, however, the immune change was not shown to mediate the functional change, for example by Treg depletion or gamma-delta T-cell reconstitution, so the causal step remains untested [SCI-EXP] [HYP]. A defined chain that has been reported but not dissected: Akkermansia, dendritic cells and IL-17. A study of FMT in an SCI model reported that enrichment of Akkermansia species was associated with metabolic reprogramming of dendritic cells, reduced gamma-delta T-cell-derived IL-17 production, attenuated neuroinflammation and improved functional recovery (38) [SCI-EXP]. Because the whole sequence was reported in a single model and has not been replicated in an independent laboratory, we label it [SINGLE], and we note that the causal contributions have not been separated: Akkermansia mono-colonization, dendritic-cell-specific manipulation, or IL-17 neutralization have not been tested in this context. Akkermansia muciniphila should not be treated as a uniformly beneficial organism, because its effects on mucosal immunity are context dependent (18) [EXT]. Toll-like receptor and inflammasome signaling. TLR and NLRP3 signaling amplifies inflammation within the injured spinal cord and is a well-supported therapeutic target in SCI (56) [SCI-EXP]. The specific claim that gut-derived microbial ligands drive this signaling after SCI is, however, indirect: the evidence cited for TLR-mediated NLRP3 activation comes from human monocytes and not from an SCI or gut-origin design (56) [EXT], and no SCI study has shown that gut-derived ligands reach the lesion in sufficient quantity to initiate the cascade. We present gut-derived TLR/NLRP3 activation as a hypothesis [HYP] and note that the therapeutic relevance of NLRP3 inhibition in SCI does not depend on its microbial origin. One citation in this section required correction. The statement that “administration of the probiotic VSL#3 enhanced Treg populations and improved motor recovery” is often attributed to a study of TLR signaling in human monocytes; that study does not address probiotics, so the claim is unsupported as cited. The relevant primary evidence is the mouse SCI study described in Section 4.1, in which VSL#3 increased regulatory T cells at the lesion and improved locomotor recovery (10) [SCI-EXP], together with the subsequent rat cervical contusion study that found no locomotor benefit from the same preparation (17) [SCI-EXP] [INCONSISTENT]. Both are now cited, and this section reports the discrepancy rather than a single outcome.

### 5.3 Neural pathways

#### 5.3.1 Vagal and autonomic signaling

Vagal afferents transmit information from the gut to the central nervous system and participate in inflammatory reflexes. The experiments that established these functions used vagotomy or vagal stimulation in models of endotoxaemia, sepsis, post-operative ileus and colitis [EXT]. After SCI, the available studies have examined vagal and cardiovascular responses, gastrointestinal function and barrier integrity; we are not aware of a study demonstrating that vagal signaling directly determines motor recovery or spinal cord pathology after SCI, and the claim that vagotomy “highlights the importance of vagal afferents after SCI” is not supported by data on motor outcomes [HYP]. Vagal function after SCI must also be interpreted within a re-organized autonomic system: high-thoracic and cervical injuries interrupt descending control of sympathetic outflow and alter sacral parasympathetic function, so the axis contains a structural lesion and cannot be treated as an intact conduit (28, 59) [SCI-EXP]. Vagus nerve stimulation as a neuromodulatory strategy. VNS has measurable autonomic and cardiovascular effects after experimental SCI and improves autonomic and gastrointestinal endpoints in other settings (59) [SCI-EXP] [EXT]; whether it can enhance motor recovery after SCI has not been tested with motor function as an endpoint. VNS is sometimes proposed to “restore autonomic balance, improve gut function, and facilitate motor recovery” as though these were equivalent outcomes. We therefore separate them: the autonomic and gastrointestinal effects are supported, whereas the motor benefit is a therapeutic hypothesis requiring direct evaluation in a design that includes motor outcome measures, a sham-stimulation comparator and adequate follow-up [HYP]. The enteric nervous system. The enteric nervous system is a large and largely autonomous neural network that regulates motility, secretion and mucosal immunity, and it is legitimately described as a key interface; the popular epithet “second brain” adds no analytical value and is not used here. After SCI, enteric function is altered both directly, through loss of extrinsic autonomic input, and indirectly, through changes in the luminal environment; distinguishing these contributions is a prerequisite for any proposal to treat the enteric nervous system as a therapeutic target [HYP].

### 5.4 Intestinal barrier and bacterial translocation

The claim frequently made is that bacterial translocation (BT) “triggers systemic inflammatory responses that exacerbate secondary spinal cord injury and hinder motor recovery”. As set out in Section 3.3, this statement requires the separation of two propositions that are often collapsed. The first-that barrier integrity is compromised after SCI-is reasonably supported (24, 28) [SCI-EXP] [SCI-CLIN]. The second-that viable bacteria or their products translocate, and that translocation then worsens neurological outcome and motor recovery-is not. Demonstration of the second proposition requires direct detection of translocated organisms or microbial products in blood, lymph or extraintestinal organs; a temporal relationship in which translocation precedes the neurological change; and an intervention that blocks translocation and thereby alters outcome. None of the available SCI studies satisfies all three criteria. In particular, elevated circulating endotoxin is not equivalent to translocation, because plasma endotoxin assays vary in specificity and because circulating lipopolysaccharide can arise from dietary sources (29). We therefore state the relationship as follows: loss of intestinal barrier function and increased systemic inflammatory tone are associated in SCI, and gut-derived bacterial products are a plausible contributor to neuroinflammation, but whether they are a cause of impaired motor recovery is unknown [HYP]. An asymmetry should also influence study design. Interventions that restore barrier function, including probiotic supplementation, reduce gut permeability and endotoxaemia in animal models and in non-SCI patient populations [SCI-EXP] [EXT] (31); whether the resulting reduction in systemic inflammation translates into a motor benefit has not been tested with motor function as the primary outcome. The same qualification applies to the statement that the intestinal barrier is “a key therapeutic target to prevent the deleterious effects of BT on spinal cord pathology and to support motor function rehabilitation”: it is a rational target, but the functional benefit remains to be demonstrated.

### 5.5 Blood-spinal cord barrier

The blood-spinal cord barrier (BSCB) is a specialized endothelial barrier that regulates molecular and cellular exchange between the bloodstream and the spinal cord parenchyma. After SCI its disruption permits infiltration of peripheral immune cells and inflammatory mediators, amplifying neuroinflammation and tissue damage (50, 60) [SCI-EXP]. SCFAs and HDAC inhibitors are often grouped together as strategies that preserve BSCB integrity. That grouping should be separated, because the two categories of evidence are not equivalent. The pharmacological line is well supported: VPA and VPA-labeled nanoparticles increase tight junction protein expression, reduce BSCB permeability, reduce matrix metalloproteinase-9 activity and improve functional recovery after SCI (50, 51, 60) [SCI-EXP, pharmacological]. These are HDAC inhibitors, but neither is a microbial metabolite. The microbiota-derived line is thinner: butyrate is genuinely microbially produced and is an HDAC inhibitor, and its administration improves barrier-related outcomes in some SCI models (45, 48) [SCI-EXP], but the concentrations used are pharmacological rather than fermentative, and no study has shown that increasing endogenous SCFA production through the microbiota produces an equivalent effect on BSCB integrity. We therefore present BSCB protection by HDAC inhibition as an established pharmacological strategy and BSCB protection by microbial metabolites as a hypothesis [HYP]. The conclusion that follows is narrower than the original: stabilizing the BSCB is a credible therapeutic objective in SCI, and HDAC inhibition is one route to it. Whether the microbiota offers a practical second route remains to be demonstrated.

## 6 Evidence from microbiota-targeted interventions

### 6.1 Pre-clinical evidence: a structured appraisal

The pre-clinical evidence in this field is frequently described as “robust”, and FMT is said to have “demonstrated consistent benefits across multiple research groups”. The second statement is defensible for FMT; the first is not, and we do not use it. Table 1 sets out the available studies by intervention, model, comparator, reported functional outcome and principal limitation, and labels the level of evidence for each. Two patterns are evident. First, the amount of evidence differs greatly between interventions: FMT has been studied in several independent laboratories, probiotics in a handful of studies with discordant results, and prebiotics, antibiotics, metabolite supplementation and bacteriophages in few studies or none, often with motor outcome as a secondary measure. Second, the reporting of design features that determine whether a result can be interpreted-sample size per group, blinding of outcome assessment, pre-specified primary endpoint, time points, and statistical testing-is frequently incomplete. We have therefore indicated in the table and in the text when the primary source does not report a given parameter rather than omitting the parameter. This section must also avoid conflating interventions that differ in kind. Direct SCFA supplementation was discussed alongside FMT, probiotics and prebiotics, while bacteriophage therapy was described as an innovative frontier; the result was that the reader could not tell which studies support a microbiota-mediated effect and which support a metabolite-mediated or purely pharmacological one. We now separate three categories: interventions that modify the microbial community (probiotics, prebiotics, dietary fiber manipulation, FMT, antibiotics, bacteriophages); interventions that deliver metabolites or their analogs directly (sodium butyrate, other SCFAs, and pharmacological HDAC inhibitors such as VPA, which as noted in Section 5.1 is not a microbial metabolite); and interventions that remain speculative (phage therapy for motor recovery, engineered consortia, and CRISPR-edited strains). Within each category we state the level of available pre-clinical evidence, and we distinguish interventions for which a functional effect has been reproduced by independent groups (FMT) from those for which it has not (probiotics) and those for which it has not been assessed at all (phages). Finally, one citation widely used in this section does not support the statement for which it is cited. The claim that FMT could modulate the post-SCI microenvironment is sometimes attributed to a reference on bacteriophage cocktails in an *ex vivo* colon model. The relevant primary sources are the SCI FMT studies now cited (15, 16, 38) [SCI-EXP]. The corresponding SCI-specific literature on gut dysbiosis and its manipulation in animal and human SCI has been reviewed elsewhere and is used here for orientation rather than as primary evidence (21, 61).

### 6.2 Clinical evidence in people with spinal cord injury

Clinical evidence in people with SCI is sparse; the familiar description of it as “limited and predominantly observational” is accurate but does not convey what the available studies can and cannot support. Table 3 lists the identified human studies with the population, design, sample size, intervention, primary endpoint, and whether any motor outcome was measured. Three observations follow from that table. First, no study has evaluated a microbiota-targeted intervention in people with SCI with motor function as the primary endpoint. The available interventional evidence addresses gastrointestinal and urinary outcomes. The largest study is a randomized, placebo-controlled trial of a *Lactobacillus casei* Shirota drink in 164 patients with SCI, which reduced the incidence of antibiotic-associated diarrhea and was neither designed nor powered to assess motor function (62) [SCI-CLIN]. A two-case report described reduced urinary inflammatory cytokines after *Lactobacillus* administration in patients with neurogenic bladder and urinary tract infection, with no locomotor assessment (63) [SCI-CLIN]. A published crossover protocol proposes probiotic and prebiotic supplementation in elite para-athletes with feasibility (recruitment and compliance) as the endpoint (64). A cross-sectional study in Chinese Han patients with SCI identified associations between gut microbiota composition and lymphocyte subsets but did not examine motor recovery (65) [SCI-CLIN]. Second, the observational literature supports association, not causation. Cross-sectional comparisons show that community composition differs between people with SCI and controls and correlates with injury severity, bowel dysfunction and inflammatory markers (11, 12, 65) [SCI-CLIN]. Because diet, bowel-management regimens, antibiotics, proton-pump inhibitors, analgesics, activity level and time since injury all differ systematically between groups, these correlations cannot be interpreted as evidence that dysbiosis impairs recovery from SCI. Third, the statement that there is an “absence of RCTs with motor function as the primary outcome” requires a defined scope. We state it precisely: among randomized controlled trials of microbiota-targeted interventions (probiotics, prebiotics, dietary interventions, FMT, antibiotics, or metabolite supplementation) conducted in people with SCI, none has used a validated motor outcome as its primary endpoint. We do not claim that no such trial exists in other populations, nor that motor outcomes were never recorded as secondary measures. This narrower formulation is what the evidence supports; the broader claim is not supported by the scope of our search [SCI-CLIN]. The most important consequence is a design consequence rather than a rhetorical one. Because motor recovery is not captured by the gastrointestinal endpoints that have been studied, and because the confounding structure of the observational data is strong, progress requires multicenter randomized trials in people with SCI that specify a validated motor outcome-for example the ISNCSCI motor score, the Walking Index for Spinal Cord Injury (WISCI II), or the Spinal Cord Independence Measure (SCIM)-as a pre-specified primary endpoint, that stratify by injury level and completeness, and that record medication, diet and bowel-management covariates. This is stated as an explicit priority in Section 8.

**Table 3:** Human studies of microbiota-targeted interventions and microbiota profiling in spinal cord injury.

Study	Population	Design	n	Intervention	Primary endpoint and main result	Motor outcome assessed?
Wong et al. (62)	Adults with SCI	Randomized, placebo-controlled trial (single center)	164	*Lactobacillus casei* Shirota probiotic drink	Antibiotic-associated diarrhea: reduced incidence (17.1 vs. 54.9%, *P* < 0.001)	No; not assessed
Anukam et al. (63)	Two patients with SCI, neurogenic bladder and UTI	Case series	2	*L. rhamnosus* GR-1 plus *L. reuteri* RC-14	Urinary cytokine concentrations: decreased TNF-alpha, IL-6 and IL-8 in the treated patient	No; not assessed
Glisic et al. (64)	Elite para-athletes (study protocol)	Crossover randomized controlled trial protocol	-	Probiotics plus prebiotics	Feasibility: recruitment and compliance	No; not an outcome
Pang et al. (65)	Chinese Han patients with SCI	Cross-sectional observational	Not reported in the abstract	None (microbiota profiling)	Association between gut microbiota composition and lymphocyte subsets	No; not assessed
Zhang et al. (11)	Men with chronic complete SCI	Cross-sectional observational	Not applicable	None (microbiota profiling)	Microbiota composition differs from controls and correlates with bowel dysfunction and inflammatory markers	No; not assessed
Bazzocchi et al. (22)	Patients with acute SCI	Prospective observational	Not applicable	None (microbiota profiling)	Compositional change in the acute phase correlates with lesion severity	No; not assessed

UTI, urinary tract infection; ISNCSCI, international standards for neurological classification of spinal cord injury; WISCI II, Walking Index for Spinal Cord Injury II; SCIM, spinal cord independence measure.

### 6.3 Rehabilitation and the microbiota: interaction rather than synergy

The term “rehabilitation-microbiota synergy” is widely used in this field. We do not use it, because synergy is a specific claim-that the combined effect exceeds the sum of the individual effects-and it has not been tested. What can be stated is weaker and more useful: rehabilitation is one of the few interventions in this field already delivered at scale, and it may both change the microbiota and be influenced by it. Distinguishing the two directions is essential. Direction 1: rehabilitation changes the microbiota. Physical activity and training alter gut microbial composition and metabolic output in humans and animals [EXT] (66), and this is plausible in SCI through changes in transit time, body composition, dietary intake and autonomic tone. Evidence in SCI is limited. An exoskeleton-assisted gait training review proposes that part of the benefit of such training may be mediated through the microbiota-brain axis (66); this is a hypothesis, and the review does not provide the mechanistic evidence that would establish it [HYP]. Electroacupuncture and related interventions have been reported to modulate the microbiota and inflammatory responses in SCI models, but the studies do not separate the microbiota-dependent component from the direct neurophysiological effect of stimulation [SCI-EXP] [HYP]. Direction 2: the microbiota modifies the response to rehabilitation. This is the claim with therapeutic implications, and it has not been tested. No study has shown that the microbiota determines the magnitude of the functional response to a given dose of training, and no trial has randomized participants to microbiota modification before rehabilitation with a functional endpoint. A negative result deserves emphasis, because it is directly relevant to the framing of this section. In a study published in Gut Microbes, rehabilitation did not restore SCI-associated gut dysbiosis (67) [SCI-CLIN]. This is an important finding: it shows that rehabilitation and microbiota modification are not interchangeable and that the effect of training on the community should not be assumed. It also raises testable questions about the dose, timing, type and duration of training. We therefore present the rehabilitation-microbiota relationship as an interaction requiring formal testing, rather than as a synergy that can be assumed.

## 7 Clinical translation challenges

### 7.1 Individual variability and heterogeneous treatment response

Inter-individual variability in gut microbiota composition is real, is large, and is a genuine obstacle to translation: the same intervention produces different compositional and metabolic responses in different hosts. The question is which analytical tools can usefully address it. 16S rRNA-based enterotype classification and machine-learning prediction of therapeutic response are frequently presented as tools for stratification and dynamic treatment adjustment. Both require qualification. Enterotype classification describes community structure; it does not predict treatment response, and the reproducibility and biological utility of enterotypes as discrete categories is itself debated. Stratification models that predict response have been developed mainly in irritable bowel syndrome, inflammatory bowel disease and metabolic disease, where both the response definition and the intervention differ from those in SCI; whether any such model transfers to SCI has not been tested (68) [EXT]. Machine-learning approaches in microbiome research are promising but are limited by small sample sizes, batch effects and over-fitting, and they require external validation before they can inform clinical decisions (68). We therefore distinguish three tiers: tools validated for the relevant outcome (none, for motor recovery after SCI); tools validated in other conditions that could be adapted (16S or metagenomic profiling, and multi-omics classifiers developed elsewhere); and proposals that remain prospective (dynamic, model-guided adjustment of therapy during treatment). Two design consequences follow. First, observational cohorts with pre-treatment sampling, standardized intervention and a validated motor outcome are needed before prediction is attempted; without them, classifier development would be built on outcome definitions that do not match the clinical question. Second, longitudinal sampling is necessary because post-SCI community composition is non-stationary, particularly in the acute and subacute phases, and a single baseline sample may be a poor summary of the exposure that matters. Comparable methodological experience in other conditions, including the use of multi-omics profiling for biomarker discovery in non-SCI nervous system injury, indicates that validation in an independent cohort is the rate-limiting step rather than model construction (69) [EXT].

### 7.2 Optimal timing and treatment duration

Several specific timeframes are frequently quoted in this field-a therapeutic window of 1–3 days post-SCI, treatment courses exceeding 8 weeks, regimens of less than 2 weeks, and 2–4 weeks for microbiota reconstitution after FMT-but the evidence behind each is rarely specified. We separate them below by source and by status. The 1–3 day window derives from pre-clinical work in which intervention during the acute inflammatory phase produced the largest effect on secondary injury; it reflects the biology of the acute inflammatory cascade rather than a measured human window [SCI-EXP, pre-clinical] (10, 15). Its clinical transferability is limited by precisely the factors that define the acute phase in humans: the need for surgical decompression and stabilization, the routine use of perioperative antibiotics, and the instability of patients with high cervical injuries. In people with SCI the appropriate timing has not been established, and the concept is better expressed as a hypothesis about the acute window than as a recommendation. Courses of more than 8 weeks and of fewer than 2 weeks are often presented as alternatives with different risks, but no dose-duration study in SCI supports either figure. What can be said is that duration interacts with intervention class: antibiotics act within days and their adverse effects accumulate with duration, whereas community-restoring interventions such as FMT require weeks to establish, and the trajectory of engraftment in non-SCI populations indicates that early colonization does not predict durable persistence (30) [EXT]. Neither statement establishes an optimal duration. The claim that FMT typically requires 2–4 weeks to achieve reconstitution while probiotics modulate metabolic output within days is a mechanistic expectation rather than a reported finding in SCI and should be treated as such [HYP]. Table 4 lists the parameters that remain to be determined; timing and duration are presented as open design parameters requiring clinical investigation rather than as established standards. Relevant experience in analogous clinical situations, such as the use of microbiological and immunological enteral nutrition during prolonged antibiotic therapy, suggests that the value of such interventions depends critically on when they are started and for how long they are continued (70) [EXT].

**Table 4:** Key translational challenges for microbiota-targeted therapies in spinal cord injury, with the current evidence status of each proposed strategy.

Challenge	Specific issue	Consequence for translation	Proposed strategy	Evidence status
Causality not established	The chain from a microbial or metabolite change to a functional improvement has rarely been tested within a single SCI study	Interventions may be tested clinically without evidence that the intended mechanism operates	Designs with mediation testing: defined manipulation with a defined functional outcome, plus depletion and reconstitution controls	Not addressed in most current studies [HYP]
Microbiome heterogeneity	Inter-individual variability driven by genetics, diet, medication and environment	Variable response; single-strain or single-community approaches generalize poorly	Pre-specified baseline profiling with pre-registered stratification hypotheses; longitudinal sampling	Candidate approach; not validated for response prediction in SCI [EXT]
Optimal timing	The acute window is defined in pre-clinical models only; clinical transfer is constrained by surgery and perioperative antibiotics	The window may be missed, or may conflict with standard acute care	Time-stratified designs; biomarker-guided timing; explicit recording of antibiotic exposure	Pre-clinical evidence only [SCI-EXP]
Treatment duration	No dose-duration study in SCI; duration interacts with intervention class	Claims of superiority for longer or shorter courses cannot be tested	Pre-specified duration with adequate follow-up; duration as a randomized factor	Unresolved [HYP]
Safety	Probiotic-associated invasive infection in susceptible hosts; pathogen transmission by FMT; antibiotic-associated dysbiosis	Patient harm; regulatory barriers to adoption	Pre-specified monitoring parameters; independent data safety monitoring with stopping rules; standardized donor screening	Demonstrated in other populations [EXT]; not characterized in SCI
Interspecies and model differences	Rodent gut communities and physiology differ substantially from those of humans	Limited extrapolation of pre-clinical efficacy to patients	Humanized gnotobiotic models and organoid co-culture for mechanism, with explicit labeling of extrapolation	Methodological advance, not efficacy evidence [EXT]
Concomitant medications	Antibiotics, proton-pump inhibitors, opioids and anticonvulsants alter the microbiota	Confounded assessment of intervention effects	Measure, stratify and report; medication-stratified randomization	Design requirement
Non-standardized interventions	Strain identity, viability, preparation, dose and storage vary between studies	Results cannot be compared or pooled	Harmonized standard operating procedures and minimum reporting standards	Prerequisite for cumulative science
Outcome measurement	No agreed motor endpoint for microbiota trials in SCI	Meta-analysis and regulatory assessment are impeded	Core outcome set based on ISNCSCI motor score, WISCI II and SCIM; pre-specified primary endpoint	Proposed; requires consensus

### 7.3 Safety evaluation

Safety is the constraint most likely to limit clinical adoption, and it requires a distinction that the literature often blurs: between risks observed in clinical studies and risks inferred from pre-clinical data, from other patient populations, or from mechanism. We separate them below. Observed clinical risks. In patients with SCI, the largest randomized experience is with *Lactobacillus casei* Shirota, which reduced antibiotic-associated diarrhea without a reported excess of serious adverse events (62) [SCI-CLIN]. In other populations, probiotic administration has been associated with invasive infection in immunocompromised and critically ill patients, including bacteraemia attributable to the administered organism (71) [EXT], and a randomized trial of probiotic prophylaxis in predicted severe acute pancreatitis reported increased mortality in the probiotic group (72) [EXT]. These are relevant considerations after SCI because immune dysregulation is common and because central venous access, urinary catheters and pressure injuries create portals for infection. FMT carries the risk of transmitting pathogens, including multidrug-resistant organisms, and the largest long-term safety datasets derive from recurrent C. difficile infection rather than from SCI (41, 42) [EXT]. Inferred or uncertain risks. The statement that prolonged antibiotic use can induce “irreversible dysbiosis” is too categorical. The magnitude and reversibility of antibiotic-induced change depend on the antibiotic, its spectrum and duration, host characteristics and the availability of a reservoir for recolonisation; recovery is often partial and slow rather than absent, and in some individuals the community does not return to its pre-treatment state (14) [SCI-EXP] [EXT]. The term is therefore qualified accordingly throughout this review (Section 4.3). Two further points follow. First, an explicit statement is needed of which parameters are monitored-infection incidence and attributable organisms, metabolic and hepatic indices, gastrointestinal bleeding, and immune and inflammatory markers-because without pre-specified monitoring, adverse events cannot be attributed to the intervention. Second, independent data safety monitoring is a design requirement rather than a recommendation, and given the uncertainty in this field the monitoring committee should be empowered to stop a trial on predefined safety criteria. We have retained the recommended framework-microbial resistance surveillance, metabolomic profiling including serum endotoxin, and immunological markers-but now present it as a monitoring proposal with the specific assays to be defined in each protocol, rather than as an existing standard [HYP]. Because the absolute risk of harm in this population is unknown, the appropriate design for a first-in-SCI trial is a small adequately monitored study with predefined stopping rules rather than an efficacy trial.

### 7.4 Standardized translational pathways

The obstacles to translation in this field are of two different kinds, and conflating them leads to inflated expectations. The first concerns the relevance of pre-clinical models: rodent gut communities differ substantially from human communities in composition and metabolic capacity, animals are typically young, male, specific-pathogen-free and housed under conditions that differ from human life, and comorbidity and polypharmacy are absent. Humanized gnotobiotic animals colonized with human-derived microbiota, and organoid-microbe co-culture systems, increase the human relevance and internal reproducibility of experiments and are therefore valuable (73, 74) [EXT]. They do not, however, predict therapeutic efficacy in patients with SCI, and it is important to state explicitly that these are models for mechanism and hypothesis generation rather than surrogates for clinical trials. Large-animal models, including porcine SCI, occupy an intermediate position and are relevant to microbiome research because they permit longitudinal sampling and clinically comparable injury, but they do not remove the need for human trials (75, 76) [EXT]. The second kind of obstacle concerns the comparability of interventions. Strain identity and viability, preparation and storage, dose, route and duration differ between studies, and without harmonization results cannot be aggregated or compared. Standard operating procedures covering strain characterization and genome sequencing, viability assays, dosing, storage and reporting are a prerequisite for cumulative science and for regulatory review (73, 75) [EXT]. A third kind of obstacle is clinical rather than experimental. Patients with SCI frequently receive antibiotics, proton-pump inhibitors, opioids and anticonvulsants that alter the microbiota and confound the assessment of a microbiota-directed intervention; dietary intake and bowel-management regimens vary widely; and the heterogeneity of injury level, completeness and time since injury is large. These variables do not disappear in a trial, so they must be measured, stratified and reported rather than treated as nuisance. Table 4 summarizes the resulting strategy. We have deliberately retained the recommendation of integrative translational frameworks while removing the implication that better models or better protocols will by themselves demonstrate clinical efficacy.

## 8 Future research directions

### 8.1 Precision microbiota interventions based on multi-omics integration

Multi-omics approaches-metagenomics, metatranscriptomics, metabolomics and proteomics-can characterize the post-SCI microbiome and its metabolic output in far greater detail than 16S profiling, and they are the most plausible route to identifying the organisms and metabolites worth targeting. It is essential, however, to be clear about what such studies produce. Metagenomic and metabolomic analyses generate associations: they identify taxa and metabolites that covary with an outcome. They do not identify causal agents, and targeting a taxon that is depleted after SCI may be ineffective if the depletion is a consequence rather than a cause. It is often stated that lower abundances of SCFA-producing bacteria such as *Faecalibacterium* and *Butyricimonas* “correlate with impaired locomotor recovery” and that integrating multi-omic data “can pinpoint key pathogenic bacterial taxa and beneficial metabolites, enabling targeted interventions”. The first half of that statement is supported, in SCI models and clinical samples, by associations between SCFA-producing taxa or SCFA concentrations and functional or inflammatory markers (15, 16) [SCI-EXP] [SCI-CLIN], although the reference most frequently cited for it reports a study of the gut-muscle axis in older adults with no locomotor outcome. The second half of the statement is a hypothesis: association-guided targeting has not yet produced a validated intervention in SCI [HYP]. The methodological requirements for progress are therefore conventional but demanding: prospectively collected, longitudinally sampled cohorts with standardized clinical covariates; sample sizes adequate for the dimensionality of the data; external validation of any predictive model; and interventional confirmation. A taxon or metabolite implicated by multi-omics should be regarded as a candidate requiring experimental validation in an SCI model and subsequent controlled testing, not as a target. Genome editing of gut bacteria, for example to increase butyrate production, and colon-targeted delivery systems are legitimate precision-medicine proposals (77) [EXT], but they currently rest on engineering feasibility demonstrated outside SCI, and they carry questions of biocontainment, ecological stability and regulation that are at least as demanding as their construction [HYP]. Table 5 lists the directions that we consider most likely to change the field, together with the evidence that would be required to move each from proposal to practice.

**Table 5:** Future research directions for microbiota-targeted therapy in spinal cord injury, with the evidence that would be required to move each direction from proposal to practice.

Research direction	Key approach	What would constitute evidence of success	Priority
Causal mediation in SCI models	Defined microbial or metabolite manipulation with mediation testing (depletion, reconstitution, receptor blockade)	Demonstration that the functional effect is abolished when the proposed mediator is removed	Critical
Randomized trials with motor endpoints	Multicenter, double-blind, microbiome-stratified design with the ISNCSCI motor score, WISCI II or SCIM as the pre-specified primary endpoint	Statistically significant improvement in a validated motor outcome with adequate safety data	Critical
Rehabilitation-microbiota interaction	Factorial or fractional factorial design with a standardized, monitored training dose and planned interaction testing	A quantified interaction effect (supra-additive, additive or negative) between the two interventions	High
Multi-omics-guided candidate identification	Longitudinal cohorts with standardized covariates; external validation of predictive models	A candidate taxon or metabolite that survives interventional testing in an SCI model and a controlled human study	High
Biomarkers of treatment response	Prospectively tested, pre-specified stratification hypotheses	Prospective prediction of response in an independent cohort of patients with SCI	High
Donor selection and engraftment metrics	Strain-resolved tracking in FMT studies; explicit donor eligibility criteria	Demonstrated donor-strain persistence that is linked to a functional outcome	Medium
Engineered microbes and delivery systems	CRISPR-edited SCFA-producing strains; colon-targeted delivery	Efficacy and biocontainment demonstrated in an SCI model	Medium
Phage therapy for defined taxa	Selective depletion of a specified pathogenic taxon with motor outcome measurement	Selective depletion with functional benefit and preservation of community diversity	Medium
Novel neuroimmune targets	Vagal modulation; endogenous progenitor activation; single-cell and spatial profiling	A cell-type-specific mechanism demonstrated together with a functional endpoint	Medium

### 8.2 Combined microbiota and rehabilitation strategies

Combining microbiota-directed interventions with rehabilitation is attractive because the two act through different mechanisms and because rehabilitation is already part of standard care. Two specific claims that are frequently made require correction. First, the statement that probiotics combined with exercise training produced “approximately 50% faster motor function recovery compared to single interventions” traces to a study of fecal microbiota transplantation in Alzheimer's disease. That study did not use an SCI model, did not include an exercise intervention, and did not measure motor recovery as defined here; the figure cannot be substantiated and is not reported here. Even in other neurological conditions, transferring microbiome findings to clinical practice requires condition-specific trials rather than extrapolation from one disease to another (78) [EXT]. What can be stated is weaker: in rodent models, exercise training and microbiota-directed interventions each modulate neurotrophic factor expression and inflammatory tone, and in non-SCI populations exercise training alters gut microbial composition and metabolic output (66, 79) [EXT]. Whether the combination produces a supra-additive effect on motor recovery after SCI has not been tested [HYP]. Second, the claim that electrical stimulation and microbiota modulation “may exert complementary effects” by acting centrally and peripherally is a mechanistic prediction with no supporting experiment in SCI. Neuromodulation, including epidural spinal cord stimulation and transcranial magnetic stimulation, has established motor effects in selected patients with SCI; its interaction with the gut microbiome has not been examined. We therefore propose a specific and feasible study design rather than a general appeal to synergy: a factorial randomized trial in which participants with SCI are assigned to microbiota-directed intervention or control and to a standardized rehabilitation dose or control, with a validated motor outcome as the primary endpoint, pre-specified interaction testing, and factorial analysis capable of distinguishing additive from supra-additive effects. Two design features are essential. The rehabilitation dose must be standardized and monitored, because it is a treatment as much as the microbiota intervention is. And interaction must be tested rather than assumed, which requires a sample size planned for the interaction term rather than for main effects alone.

### 8.3 Novel targets in the neuroimmune-microbiota axis

Two candidate targets in the neuroimmune-microbiota axis merit attention, and both are currently hypotheses. The vagal pathway. Vagal afferents can influence central inflammatory responses, and in SCI vagal stimulation also has measurable autonomic and cardiovascular effects (59) [SCI-EXP]. The proposal that vagal modulation can directly attenuate spinal cord inflammation and thereby support motor recovery has not been tested in an SCI model with both inflammatory and motor endpoints, and it should be evaluated as such. Practical requirements include a motor outcome measure, a sham-stimulation control, and separate reporting of autonomic and inflammatory endpoints so that any effect on motor function can be distinguished from a nonspecific systemic effect [HYP]. Endogenous spinal cord stem and progenitor cells. The statement that microbiota-derived metabolites or signaling molecules “can influence endogenous spinal cord stem cell populations... and may enhance the regenerative capacity of these cells” has no direct experimental support in SCI. Ependymal cells lining the central canal do have proliferative and differentiation potential, and modulating their behavior is a legitimate therapeutic idea, but no study has shown that a microbial metabolite changes the fate or output of these cells after SCI. This is therefore presented as a hypothesis requiring cell-level demonstration before any interventional proposal [HYP]. A third direction is methodological. Single-cell and spatial transcriptomic approaches can characterize how defined microbial interventions change microglia, astrocytes and other cell populations in the injured cord with a resolution that bulk tissue analysis cannot achieve, and this is the most plausible route to identifying cell-type-specific mediators of any microbiota effect (61) [SCI-EXP]. Used in conjunction with the graded evidence framework in Box 1, such studies would allow specific mechanistic steps-rather than whole pathways-to be confirmed or rejected.

## 9 Conclusion

The relationship between the gut microbiota and recovery after spinal cord injury is a genuine and promising research direction, but the evidence is less advanced than the tone of much of the existing literature implies. Our assessment after reviewing the field is as follows. Pre-clinical studies consistently show that SCI alters the gut community and that microbiota-directed interventions-most reproducibly FMT-can improve locomotor outcomes in rodent models. They do not, with rare exceptions, show that the microbial or metabolite change causes the functional improvement, because the causal chain has almost never been tested within a single SCI study. In humans, the evidence is observational and associative, no randomized trial has used a validated motor outcome as its primary endpoint, and the interventional studies that exist addressed diarrhea, urinary infection and feasibility rather than motor recovery. The descriptions of the pre-clinical evidence as “robust”, and of the mechanistic picture as having “clarity” sufficient to establish the microbiome as a clinically ready therapeutic target, are not supported by the evidence reviewed here, and we do not use them. The conclusion is therefore framed in the opposite direction: rather than presenting the microbiome as a clinically promising target on the strength of pre-clinical data, it states what is known and what would be required to know more. What is known: the gut-spinal cord axis is anatomically and physiologically plausible; SCI alters gut function and community composition; several proposed pathways have mechanistic support in non-SCI systems; and a small number of interventions improve functional outcomes in animal models. What is not known: whether the microbiota causally limits motor recovery in humans; whether modifying it improves motor recovery; which taxa, strains or metabolites matter; when to intervene and for how long; and how to identify people who will respond. The two claims about future trials that are commonly made in this context also require revision. We proposed stratification by microbiome profile, personalized treatment, biomarker development and therapeutic algorithms, but did not identify which microbiome characteristics can reproducibly classify responders, or which biomarkers have been validated to predict motor recovery after SCI. The honest position is that neither currently exists. Stratification variables should therefore be pre-specified on the basis of the strongest available association data and treated as an explicit hypothesis to be tested, rather than as an established method of personalization (68) [EXT]. The practical priorities follow from that position. First, generate SCI-specific causal evidence: experiments in which a defined microbial or metabolite change is manipulated and a defined functional outcome is measured, with mediation tested directly. Second, build the clinical infrastructure: multicenter randomized trials in people with SCI with a validated motor outcome as the pre-specified primary endpoint, a standardized rehabilitation dose, and systematic recording of antibiotics, diet and bowel management. Third, harmonize reporting: model, species, injury level and severity, sample size, intervention dose and viability, time points, and statistical analysis, so that results can be compared and, where appropriate, pooled. Fourth, adopt an explicit framework for the strength and provenance of evidence, as proposed in Box 1, so that findings obtained in other diseases are not transferred to SCI without verification. Achieving these steps would move the field from descriptive microbiome characterization and promising pre-clinical signal to causally informed, testable interventions. That transition is more demanding than the current enthusiasm suggests, but it is also the only route by which microbiota-directed strategies can become a credible component of rehabilitation after spinal cord injury.
